# Critical level of food insecurity and nonadherence to antiretroviral therapy among adults living with HIV

**DOI:** 10.5588/pha.25.0043

**Published:** 2026-03-06

**Authors:** N.L. Mbiki, M.S. Dimfumu, B.E. Disuemi, G.M. Ngombua, S.B. Bindamba, K.T. Totokani, G.D. Nahimana, B.P. Mutombo

**Affiliations:** 1 Department of Nutrition, Kinshasa School of Public Health, University of Kinshasa, Kinshasa, Democratic Republic of the Congo;; 2 Ministry of Public Health, National Nutrition Program (Program Directorate and Department of Surveillance and Research) and National Program for Neglected Tropical Diseases, Kinshasa, Democratic Republic of the Congo;; 3 Clinton Health Access Initiative, Kinshasa, Democratic Republic of the Congo;; 4 Helen Keller International, Kinshasa, Democratic Republic of the Congo;; 5 School of Public Health, University of Kinshasa, Kinshasa, Democratic Republic of the Congo.

**Keywords:** viral suppression, severe food insecurity, nutritional support, Kinshasa, Democratic Republic of the Congo

## Abstract

**BACKGROUND:**

Adherence to antiretroviral therapy (ART) is crucial for the clinical management of people living with HIV (PLHIV), but food insecurity can compromise it. The threshold at which food insecurity significantly affects adherence remains poorly studied.

**METHODS:**

A cross-sectional study was conducted in health care facilities in Kinshasa among 506 PLHIV receiving outpatient ART. Socio-demographic, clinical, behavioural, and nutritional data were collected through structured interviews and medical record reviews. Chi-square tests and logistic regression analyses were performed using Stata version 17.0 software, with a statistical significance threshold set at *P* < 0.05.

**RESULTS:**

Proportion of ART nonadherence was 51.4%. Overall, 74.9% of participants were food insecure, including 56.5% with severe food insecurity. In multivariate analysis, severe food insecurity was strongly associated with nonadherence (adjusted odds ratio [aOR] = 5.98). Other predictors included alcohol use (aOR = 1.93), absence of viral load monitoring (aOR = 1.63), travel outside Kinshasa (aOR = 3.52), and being widowed or divorced (aOR = 2.18). ART refill intervals of 3–6 months were protective (aOR = 0.42).

**CONCLUSION:**

Severe food insecurity significantly undermines ART adherence. Integrating targeted nutritional support into HIV programmes, alongside biomedical care, may help improve treatment adherence and support progress towards achieving the UNAIDS 95-95-95 goals.

HIV infection remains a major global public health concern. In 2023, an estimated 39.9 million people were living with HIV and 630,000 died from opportunistic infections, with sub-Saharan Africa accounting for approximately 3,100 new infections per day.^1^ In the Democratic Republic of the Congo (DRC), HIV prevalence is estimated at 1.0% in the general population and 0.4% in Kinshasa,^2^ representing more than 500,000 people living with HIV (PLHIV) nation-wide.^3^ Progress towards the UNAIDS 95-95-95 targets has been notable: by 2023, 87% of PLHIV knew their status, 98% of those diagnosed were on antiretroviral therapy (ART), and among the 46% who had viral load testing, 89% achieved suppression equivalent to an estimated 75.9% overall viral suppression.^1^ Viral suppression improves clinical outcomes and reduces sexual and perinatal transmission^4^ but relies heavily on sustained ART adherence.^5^ Adherence, defined as the degree to which patients follow prescribed regimens,^6^ is key to therapeutic success; poor adherence contributes to virologic failure, disease progression, and increased morbidity.^5,6^

Food insecurity is a well-established determinant of poor adherence. Defined as limited or uncertain access to sufficient, safe, and nutritious food,^7^ it affects health care access, quality of life,^8^ psychological well-being,^9^ hospitalisation rates, and mortality.^10^ Evidence shows a consistent association between food insecurity and ART nonadherence.^11^ However, most studies treat food insecurity as a binary exposure, neglecting potential gradient effects across mild, moderate, and severe categories.^12^ This simplification limits the ability to determine the severity threshold at which adherence begins to deteriorate. In the DRC, where food insecurity is widespread, PLHIV face heightened vulnerability, yet some maintain adequate adherence despite hardship. This suggests that nonadherence may emerge only beyond a certain severity level, underlining the relevance of exploring a dose–response relationship.^13^

This study therefore investigates the association between graded levels of food insecurity and ART adherence among PLHIV in Kinshasa. The findings aim to inform targeted nutritional and therapeutic interventions and support progress towards the national 95-95-95 goals.

## METHODS

This cross-sectional analytical study was conducted from November 2024 to January 2025 among adults living with HIV attending outpatient services in six geographically distributed health care facilities in Kinshasa, Democratic Republic of the Congo. All centres provided free ART and none offered food or nutritional assistance during the study period.

### Participants and recruitment

Eligible participants were adults (≥18 years) living with HIV, on ART for at least 6 months, and receiving routine follow-up in the selected facilities. Patients who were hospitalised or presented severe psychiatric or cognitive impairment were excluded. Consecutive recruitment was performed during routine visits after confirming eligibility and obtaining written informed consent.

### Sampling and sample size justification

The sample size was calculated at 385 using a 95% confidence level and 5% margin of error, assuming 50% nonadherence. Accounting for a 10% nonresponse rate, the final target was 511 participants. A three-stage sampling strategy was employed: first, six high-volume facilities were selected; then, adults on ART for at least 6 months were identified; and finally, consecutive enrolment occurred.

### Data collection tools and procedures

Data were collected through a structured questionnaire administered in French or Lingala and complemented by medical record review. The questionnaire, programmed in KoboCollect, included socio-demographic characteristics, food insecurity assessed using the Food Insecurity Experience Scale (FIES), adherence to ART using a composite indicator (Morisky Medication Adherence Scale [MMAS-8], AACTG, pharmacy refill, and clinical follow-up), and clinical variables (CD4 count, viral load, regimen, weight, appointment attendance).

### The primary outcome

ART nonadherence was defined as nonadherence identified through any of three sources: pharmacy refill delays (≥1 day), missed doses reported during clinical follow-up, low self-reported adherence using the AACTG^14^ and the Morisky Medication Adherence Scale (MMAS-8).^15^

### The main exposure

Food insecurity was categorised into food secure, mildly, moderately, or severely insecure based on the FIES.^16^ Covariates included socio-demographic characteristics, treatment duration, treatment line, reasons for missed doses, and appointment adherence.

### Data quality control and management

Investigators were trained for 3 days, and the tool was pretested on 10% of the expected sample in non-study sites. Daily supervision ensured completeness and accuracy. Data were entered in EpiData 3.1 and analysed in Stata 17.

### Statistical analysis

Descriptive statistics summarised participant characteristics. Chi-square tests explored factors associated with food insecurity. Variables with *P* < 0.05 in bivariate analyses were included in multivariate logistic regression. Adjusted odds ratios (aORs) with 95% confidence intervals (CIs) were reported; significance was set at *P* < 0.05.

### Ethical statement

Ethical approval was granted by the Ethics Committee of the School of Public Health, University of Kinshasa. Written informed consent was obtained from all participants.

## RESULTS

In total, 506 of the 511 adult PLHIV screened met the inclusion criteria, and they were selected for this study. Participants had a median age of 38 years (interquartile range: 18–57), were mostly male (55.1%), single (54.7%), and had at least secondary education (91.1%). Most were unemployed (74.9%) and lived in households of five or more members (68.0%). Among the 272 participants with CD4 measurements, 24.6% had counts >500 cells/mm^3^ (Table 1).

**TABLE 1.** Characteristics of adults with HIV followed in the care facilities of Kinshasa, 2024.

Characteristics	Total (*n*)	Adherence to ART		*P* value
		Yes	No	
		*n* (%)	*n* (%)	
Gender				0.001
Men	279	117 (47.6)	162 (62.3)	
Women	227	129 (52.4)	98 (37.7)	
Marital status				0.004
Single	277	133 (54.1)	144 (55.4)	
Married	133	78 (31.7)	55 (21.2)	
Divorced and widowed	96	35 (14.2)	61 (23.5)	
Level of study				0.016
No	29	8 (3.3)	21 (8.1)	
Primary	16	5 (2.0)	11 (4.2)	
Secondary	301	144 (58.5)	157 (60.4)	
Superior	160	89 (36.2)	71 (27.1)	
Respondent’s main activity				<0.001
Employee	127	66 (26.8)	61 (23.5)	
Self-employed	134	42 (17.1)	92 (35.4)	
Unemployed	245	138 (56.1)	107 (41.2)	
Household size				0.025
<5	162	67 (27.2)	95 (36.5)	
≥5	344	179 (72.8)	165 (63.5)	
Appointment adherence				0.004
Yes	448	228 (92.7)	220 (84.6)	
No	58	18 (7.3)	40 (15.4)	
ART regimen				0.009
First-line	196	96 (68.6)	100 (54.4)	
Second-line	128	44 (31.4)	84 (45.6)	
Primary caregiver				0.012
Another person	224	123 (54.9)	101 (45.1)	
Self	282	123 (43.6)	159 (56.4)	

ART = antiretroviral therapy.

### Proportion and level of food insecurity associated with nonadherence to ART among PLHIV adults, 2024

Overall, 51.4% of participants were nonadherent to ART. Food insecurity was highly prevalent (74.9%), with 56.5% experiencing severe and 18.4% moderate insecurity; only 25.1% were food secure (Figure; Table 2). Both moderate (odds ratio [OR] = 2.35; 95% CI: 1.28–4.32) and severe food insecurity (OR = 9.65; 95% CI: 5.82–15.99) were significantly associated with increased odds of nonadherence.

**FIGURE.**
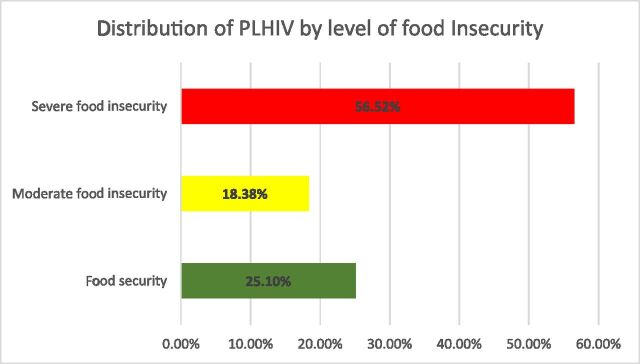
Level of food insecurity associated with nonadherence to antiretroviral therapy.

**TABLE 2.** Proportion and level of food insecurity associated with nonadherence to ART among PLHIV adults enrolled in HIV care in Kinshasa health care facilities.

Variables	Frequency, *n* (%)	cOR (95% CI)
Food insecurity	379 (74.8)	
Level of food insecurity		
Food security	127 (25.1)	r
Moderate	93 (18.38)	2.35 (1.28–4.32)
Severe	286 (56.52)	9.65 (5.82–15.99)

ART = antiretroviral therapy; PLHIV = people living with HIV; cOR = crude odds ratio; CI = confidence interval.

### Factors associated with nonadherence to ART among PLHIV

In univariate analysis, nonadherence was associated with several socio-demographic, behavioural, and treatment-related characteristics (Table 3). Lower odds of nonadherence were observed among women (OR = 0.55; 95% CI: 0.38–0.78) and among participants on ART for 3–6 months (OR = 0.43; 95% CI: 0.29–0.63). Higher odds were observed among single participants (OR = 1.53; 95% CI: 1.01–2.33) or divorced participants (OR = 2.47; 95% CI: 1.44–4.24), those without formal education (OR = 3.29; 95% CI: 1.38–7.87), self-employed individuals (OR = 2.37; 95% CI: 1.43–3.92), alcohol users (OR = 3.45; 95% CI: 2.39–4.97), drug users (OR = 2.91; 95% CI: 1.98–4.26), or those missing clinic appointments (OR = 2.30; 95% CI: 1.28–4.14), practicing self-management of ART (OR = 1.57; 95% CI: 1.11–2.24), or travelling outside Kinshasa (OR = 2.83; 95% CI: 1.86–4.30). Moderate and severe food insecurity were among the strongest predictors.

**TABLE 3.** Factors associated with nonadherence to ART among adult PLHIV receiving HIV care in Kinshasa health care facilities, 2024.

Characteristics	Total	Univariate analysis		Multivariate analysis	
		cOR	95% CI	aOR	95% CI
Gender					
Men	279	r			
Women	227	0.55**	0.38–0.78		
Age (years) median (IQR) 38 ± 19					
30–39	156	r			
<30	113	0.84	0.52–1.36		
40–49	114	0.92	0.57–1.49		
50–59	88	0.79	0.47–1.34		
≥60	35	0.90	0.43–1.87		
Marital status					
Married	133	r			
Single	277	1.53*	1.01–2.33	1.38	0.79–2.40
Divorced and widowed	96	2.47**	1.44–4.24	2.18*	1.10–4.35
Level of study					
Superior	160	r			
No	29	3.29**	1.38–7.87		
Primary	16	2.75	0.92–8.30		
Secondary	301	1.37	0.93–2.01		
Main activity					
Employee	127	r			
Self-employed	134	2.37**	1.43–3.92		
Unemployed	245	0.83	0.55–1.29		
Duration of ART provided					
<3 months	209	r			
3–6 months	210	0.43***	0.29–063	0.42**	0.25–0.69
>6 months	87	1.05	0.63–1.75	1.30	0.65–2.58
Viral load test done					
Yes	230	r			
No	276	2.58***	1.80–3.69	1.63*	1.03–2.58
Alcohol consumption					
No	239	r			
Yes	267	3.45***	2.39–4.97	1.93*	1.07–3.47
Drug use					
No	325	r			
Yes	181	2.91***	1.98–4.26		
Travel outside Kinshasa					
No	371	r			
Yes	135	2.83***	1.86–4.30	3.52***	2.03–6.11
Keeping your appointment					
No		r			
Yes		2.30**	1.28–4.14		
Support					
Third party		r			
Yourself		1.57*	1.11–2.24		
Level of food insecurity					
Food security	127	r			
Moderate	93	2.35**	1.28–4.32	1.64	0.83–3.24
Severe	286	9.65***	5.82–15.99	5.98***	3.35–10.69

*P < 0.05, **P < 0.01, ***P < 0.001.

r = reference category; ART = antiretroviral therapy; PLHIV = people living with HIV; cOR = crude odds ratio; aOR = adjusted odds ratio; CI = confidence interval; IQR = interquartile range.

In multivariate analysis, severe food insecurity remained the strongest independent predictor of nonadherence (aOR = 5.98; 95% CI: 3.35–10.69; *P* < 0.001). Additional independent predictors included travel outside Kinshasa (aOR = 3.52; *P* < 0.001), widowed/divorced status (aOR = 2.18; *P* = 0.025), alcohol consumption (aOR = 1.93; *P* = 0.028), and absence of viral load monitoring (aOR = 1.63; *P* = 0.035). ART duration of 3–6 months remained protective (aOR = 0.42; *P* = 0.001).

## DISCUSSION

This study examined the association between food insecurity and ART adherence and identified the severity level at which food insecurity significantly reduces adherence among PLHIV. More than half of the participants (51.38%) were nonadherent, a level that threatens progress towards the UNAIDS 95-95-95 targets, as poor adherence increases the risk of drug resistance and virologic failure.^5,17,18^ This prevalence is higher than that previously reported in the DRC (25%–30%),^19^ possibly due to differences in adherence measurement or the recent deterioration of socio-economic conditions in Kinshasa.

Food insecurity was highly prevalent (74.9%), with 56.52% experiencing severe food insecurity. This burden adversely affects immunity, mental health, and overall disease management, and is consistently linked to poor adherence in sub-Saharan Africa.^20^ Our findings therefore align with existing evidence showing that poverty and unstable access to food undermine HIV outcomes. After adjustment, severe food insecurity remained the strongest predictor of nonadherence. This gradient-based analysis represents a methodological improvement compared with binary classifications used in previous studies. Several mechanisms may explain this association: from biological, some ART regimens cause gastrointestinal or neurological side effects when taken without food,^21,22^ and nutritional recommendations supporting ART effectiveness may be unattainable in food-insecure settings.^23^ From psychosocial, chronic hunger increases stress, anxiety, and depression, which impair treatment consistency.^24^ From economic, limited financial resources may force patients to prioritise food over transport or medical costs, reducing continuity of care.^25^

In the DRC, where armed conflict, political instability, and food price inflation persist, PLHIV remain especially vulnerable.^26^ The magnitude of the association observed in our study exceeds that described in other regions, where severe food insecurity generally doubled the risk of nonadherence.^27^ This difference may reflect heightened economic vulnerability in Kinshasa or contextual barriers to stable access to care. Other determinants of nonadherence were also identified. Lack of viral load monitoring may signal irregular follow-up, depriving patients of reinforcing feedback when viral suppression is achieved. Alcohol consumption, a known barrier to adherence due to forgetfulness and reduced judgement,^28^ may additionally reflect underlying mental health concerns. Travel outside Kinshasa was associated with missed doses and disrupted follow-up, underscoring the need for extended refills and interprovincial ART coordination.^27^ Treatment duration between 3 and 6 months was protective, while refills exceeding 6 months increased nonadherence, possibly due to treatment fatigue or perceived recovery.^29,30^ Widowhood and divorce also emerged as vulnerability factors, consistent with findings from southern Africa.

This study provides the first quantification in the DRC of the critical food insecurity threshold affecting ART adherence. Strengths include methodological rigour, a validated FIES scale, a composite adherence metric, adjustment for key confounders, and a large multisite sample from both public and private facilities. However, generalisability may be limited outside Kinshasa. Limitations include its cross-sectional design, which limits causal inference, and self-reported adherence may be biased. Bias was mitigated through triangulation with clinic appointments and biological monitoring, trained interviewers, and a composite adherence measure combining subjective and objective indicators.

## CONCLUSION

Severe food insecurity is strongly associated with ART nonadherence among PLHIV in Kinshasa. Identifying a critical threshold at which adherence declines highlights food access as a major barrier to HIV therapeutic success. These findings support Sustainable Development Goals 2 (Zero Hunger) and 3 (Good Health and Well-Being) and underscore the need for multisectoral strategies integrating nutritional support into HIV care, while informing future research aimed at comprehensive and patient-centred HIV management.
